# *Na+/K+-ATPase α-subunit* (*nkaα*) Isoforms and Their mRNA Expression Levels, Overall Nkaα Protein Abundance, and Kinetic Properties of Nka in the Skeletal Muscle and Three Electric Organs of the Electric Eel, *Electrophorus electricus*

**DOI:** 10.1371/journal.pone.0118352

**Published:** 2015-03-20

**Authors:** Biyun Ching, Jia M. Woo, Kum C. Hiong, Mel V. Boo, Celine Y. L. Choo, Wai P. Wong, Shit F. Chew, Yuen K. Ip

**Affiliations:** 1 Department of Biological Sciences, National University of Singapore, Kent Ridge, Singapore, 117543, Republic of Singapore; 2 The Tropical Marine Science Institute, National University of Singapore, Kent Ridge, Singapore, 119227, Republic of Singapore; 3 Natural Sciences and Science Education, National Institute of Education, Nanyang Technological University, 1 Nanyang Walk, Singapore, 637616, Republic of Singapore; Universidade Federal do Rio de Janeiro, BRAZIL

## Abstract

This study aimed to obtain the coding cDNA sequences of *Na^+^/K^+^-ATPase α (nkaα*) isoforms from, and to quantify their mRNA expression in, the skeletal muscle (SM), the main electric organ (EO), the Hunter’s EO and the Sach’s EO of the electric eel, *Electrophorus electricus*. Four *nkaα* isoforms (*nkaα1c1*, *nkaα1c2*, *nkaα2* and *nkaα3*) were obtained from the SM and the EOs of *E*. *electricus*. Based on mRNA expression levels, the major *nkaα* expressed in the SM and the three EOs of juvenile and adult *E*. *electricus* were *nkaα1c1* and *nkaα2*, respectively. Molecular characterization of the deduced Nkaα1c1 and Nkaα2 sequences indicates that they probably have different affinities to Na^+^ and K^+^. Western blotting demonstrated that the protein abundance of Nkaα was barely detectable in the SM, but strongly detected in the main and Hunter’s EOs and weakly in the Sach’s EO of juvenile and adult *E*. *electricus*. These results corroborate the fact that the main EO and Hunter’s EO have high densities of Na^+^ channels and produce high voltage discharges while the Sach’s EO produces low voltage discharges. More importantly, there were significant differences in kinetic properties of Nka among the three EOs of juvenile *E*. *electricus*. The highest and lowest *V*_max_ of Nka were detected in the main EO and the Sach’s EO, respectively, with the Hunter’s EO having a *V*_max_ value intermediate between the two, indicating that the metabolic costs of EO discharge could be the highest in the main EO. Furthermore, the Nka from the main EO had the lowest *K_m_* (or highest affinity) for Na^+^ and K^+^ among the three EOs, suggesting that the Nka of the main EO was more effective than those of the other two EOs in maintaining intracellular Na^+^ and K^+^ homeostasis and in clearing extracellular K^+^ after EO discharge.

## Introduction

Electric fishes possess electric organs (EOs) and are able to generate electricity through electrogenesis [1]. Strongly electric fishes produce strong electric organ discharges (EODs) for predation and defense, while weakly electric fishes produce weak EODs for electrolocation or electrocommunication [2]. The electric eel, *Electrophorus electricus* (Linnaeus), belongs to Order Gymnotiformes and Family Gymnotidae, and has a cylindrical and elongated body [3]. It can be found in the northeastern parts of South America, including the Guyanas, Orinoco Rivers and the mid to lower portions of the Amazon Basin [4]. *Electrophorus electricus* uniquely possesses three EOs and has the uncommon ability to produce both low-voltage and high-voltage EODs. A mature *E*. *electricus* can stun a prey or ward-off predators by delivering a one second burst of EOD peaking at 600 V with a current of 2 A, making it the greatest producer of bioelectricity in the animal kingdom [5]. Although the low-voltage EOD generated by *E*. *electricus* for electrolocation and communication is a mere 10 V/cm, it is still 10–100 times stronger than a typical EOD of other members of Gymnotidae [6]. When *E*. *electricus* detects prey, it positions its body into a C-shape with the prey situated at the gap, and uses high voltage EODs to immobilize it; then, it reverts to low voltage EODs to locate it.

All three EOs of *E*. *electricus* are derived from skeletal muscle, with the Sach’s EO developing first, followed by the main EO and then the Hunter’s EO [7]. These EOs are controlled by the central control nucleus in the ventral medial region of the medulla oblongata of the brain [8]. To date, little has been done on the Hunter’s organ and the Sach’s organ other than anatomy descriptions and brief reports on their abilities to generate bioelectricity [7,9]. The Sach’s EO produces low voltage EODs of about 10 V at a frequency of up to 25 Hz, while the main EO produces high voltages EODs up to 500 V at a frequency of up to several hundred Hz [10]. Both types of EODs are monophasic and last for around 2 msec [2]. Patch recordings of Sach’s EO’s electrocytes and main EO’s electrocytes show that peak currents generated by the main EO are on average double that generated by the Sach’s EO’s, which was attributed to higher electrocyte transmembrane Na^+^ channel densities in the main EO as compared to the Sach’s EO [11]. Differences in electrical discharges from the Sach’s EO and the main EO can also be attributed to physiology, more specifically, the density (and also the quantity) of electrocytes within the EOs. The main EO has more densely-packed electrocytes while the electrocytes of the Sach’s EO are spaced more distantly apart with gaps in between them nearer to the tail [9]. The Hunter’s EO, which lies ventral to both the main EO and the Sach’s EO, functions like the main EO towards the anterior end but also functions similarly to the Sach’s EO towards the posterior end [2]. Other than structural differences in electrocytes and Na^+^ channel density, there has not been any other explanation given for the different type of EODs generated by the main and Sach’s EOs, nor has there been a comparative study between all three EOs.

EOs are composed of electrocytes, which are long, multinucleated, ribbon-like cells derived from embryonic myoblasts giving rise to skeletal muscles (SMs) [12]. Electrocytes are arranged in columns which extend the length of the EO, and separated by insulating septa [9]. Each electrocyte has a flat posterior membrane, with small papillae on its surface innervated with chemical synapses, and a non-innervated rostral membrane with multiple folds [9,13]. It has a high density of transporters and channels, which are polarized to particular domains of its plasma membrane, and minimal essential organelles localized near both membranes in the cytoplasm [14]. This polarization enables electrocytes to produce transcellular potentials, the summation of which generates a powerful whole-animal EOD.

The roles of transporters and channels in facilitating ionic movements across the plasma membrane of an electrocyte are vital to electrogenesis. Voltage-gated Na^+^ channel, Cl^-^ channel, leak K^+^ channel and Na^+^/K^+^-ATPase (Nka) work in synchrony to regulate and generate transmembrane potentials [15–17]. When stimulated by neuronal signals through nicotinic acetylcholine receptors, voltage-gated Na^+^ channels, which are localized predominantly on the innervated posterior membrane, open to facilitate an influx of Na^+^, depolarizing the innervated membrane from the resting-85 mV to +65 mV [15,18]. Upon recovery, the resting potential is restored by the inactivation of voltage-gated Na^+^ channels and the operation of the Cl^-^ channels and leak K^+^ channels, while the Na^+^ and K^+^ concentration gradients are restored through the action of Nka [19].

Nka is a molecular pump that actively transports three Na^+^ out of and two K^+^ into the cell. Its main function lies in the homeostatic maintenance of Na^+^ and K^+^ concentration gradients cross the plasma membrane [20], providing the chemical potential energy for the depolarization and repolarization of the membrane voltage involved in electrocyte discharge. Nka consists mainly of α and β subunits which exist in multiple isoforms. The α and β subunits dimerize to form a functional Nka [21]. The Nkaα subunit is a large protein around 110–120 kDa, containing the Na^+^, K^+^ and ATP binding sites [22]. The Nkaβ subunit inserts and anchors the αβ complex in the plasma membrane and regulates the Na^+^ and K^+^ affinities [23]. The Nkaα subunit is highly conserved and has four isoforms (α1, α2, α3, α4) [22]. Nkaα1 is the predominant form expressed in almost all tissues and it has several sub-forms (α1a, α1b, α1c); Nkaα2 and Nkaα3 are found mainly in skeletal tissue and nervous tissue, respectively, while Nkaα4 is exclusively expressed in sperm [23,24].

Using commercially available Nkaα isoform specific anti-NKAα antibodies, Lowe et al. [25] discovered that Nkaα1 and Nkaα2 were polarized in the innervated and the non-innervated membranes, respectively, of electrocytes from the main EO of *E*. *electricus*, indicating that different Nkaα isoforms may have different physiological roles in electrogenesis. However, to date, only one *nkaα* with an unknown isoform identity has been sequenced from the electric organ of *E*. *electricus* (AF356351) [26]. Therefore, this study was undertaken to obtain the coding cDNA sequences of various *nkaα* isoforms from the SM, the main EO, the Hunter’s EO and the Sach’s EO of *E*. *electricus*, and to identify the isoform types based on their deduced amino acid sequences. Using quantitative real-time polymerase chain reaction (qPCR), efforts were made to test the hypothesis that *E*. *electricus* had a SM-predominant and an EO-predominant *nkaα*, with the latter being differentially expressed among the three EOs, and to elucidate the possible relationships between certain *nkaα* isoforms and the ability to produce strong or weak electric discharges among the three EOs. Furthermore, western blotting was performed using a commercially available anti-NKAα antibody to compare the protein abundance of NKAα among the SM and the three EOs. Finally, efforts were made to determine and compare the kinetic properties of Nka from these four tissue/organs.

### A note on gene and protein nomenclature

Two different types of abbreviations of genes/proteins have been adopted in this report, as the standard abbreviations of genes/proteins of fishes (http://zfin.org/cgi-bin/webdriver?MIval=aa-ZDB_home.apg) are different from those of human/non-human primates (http://www.genenames.org). For fishes, gene symbols are italicized, all in lower case, and protein designations are the same as the gene symbol, but not italicized with the first letter in upper case.

## Materials and Methods

### Fish

*Electrophorus electricus* (juvenile: 200–300 g, *N* = 5; adult 1800–2300g, *N* = 3) were imported from South America through a local fish farm in Singapore. No attempt was made to separate the sexes. Fish were maintained in dechlorinated tap water at 25°C and acclimated to laboratory conditions for one week. Fish were killed with an overdose of neutralized MS-222 (0.2%) followed with a strong blow to the head. Tissue samples of SM and the three EOs were excised, frozen in liquid nitrogen and stored at -80°C until analyses.

### Ethics Statement

Approval to undertake this study was granted by the Institutional Animal Care and Use Committee of the National University of Singapore (IACUC 142/12).

### Total RNA extraction and cDNA synthesis

Total RNA was extracted from muscle, main EO, Hunter’s EO and Sach’s EO samples using Tri Reagent (Sigma-Aldrich Co., St. Louis, MO, USA), and further purified using the RNeasy Plus Mini Kit (Qiagen GmbH, Hilden, Germany). Following isolation, RNA was quantified spectrophotometrically using Shimadzu BioSpec-nano (Shimadzu, Tokyo, Japan). RNA integrity was verified electrophoretically and the RNA was stored at -80°C. First strand cDNA was synthesized from 4 μg of total RNA using oligo(dT)_18_ primer and the RevertAid first strand cDNA synthesis kit (Thermo Fisher Scientific Inc.).

### Polymerase Chain Reaction (PCR)

Partial *nkaα1c1*, *α1c2*, *α2* and *α3* sequences were obtained using RACE and PCR primers (Table 1) designed on suitable regions based on multiple alignments of the *nka* sequences from various fish species available in Genbank (http://www.ncbi.nlm.nih.gov/Genbank/). PCR was performed in a Biorad Peltier thermal cycler (Biorad Laboratories, Hercules, CA, USA) using Dreamtaq polymerase (Thermo Fisher Scientific Inc.). The cycling conditions were 95°C for 3 min, followed by 40 cycles of 95°C for 30 s, 60°C for 30 s, 72°C for 2 min and a final extension of 72°C for 10 min. PCR products were separated by electrophoresis in 1% agarose gel. Bands of predicted molecular masses were excised and purified using FavorPrep Gel Purification Mini Kit (Favorgen Biotech Corp., Ping-Tung, Taiwan) according to manufacturer’s protocol. Purified PCR products were subjected to cycle sequencing using BigDye Terminator v3.1 Cycle Sequencing Kit (Life Technologies Corporation, Carlsbad, California) and sequenced using the 3130XL Genetic Analyzer (Life Technologies Corporation).

**Table 1:** Primer sequences designed for PCR, RACE-PCR and qPCR in this study.

Gene	Primer type	Primer sequence (5’ to 3’)
*nkaα1c1*	PCR	Forward (TAGGCAGGATACATCAAGG)
		Reverse (CCCTATGTGAATGAAGACTGT)
	5’ RACE-PCR	GTTTGGAGACATCAGATCCTGCGAT
	3’ RACE-PCR	CATCAACATTGCTGGGTATCCGTGTC
	qPCR	Forward (TTGTGGCTCAGTAAAGGACA)
		Reverse (CCAATGAGGGTGAGAGCAA)
*nkaα1c2*	PCR	Forward (TGAAGAAGTTGTGGTCGGT)
		Reverse (CTGAGGGCAATGAGACTGT)
	5’ RACE-PCR	CTGGTGATCGCACTGTGATGGGTCGTAT
	3’ RACE-PCR	TGTGCAGTGGGCGGATTTGATCATCTGT
	qPCR	Forward (GAGAAACTGCTGGTGATGC)
		Reverse (CACAAGAATGCAAATTCCTCAG)
*nkaα2*	PCR	Forward (ACTGCTGGCTACGGTC)
		Reverse (ATGACCTCTGAGTTCCTGG)
	qPCR	Forward (GAGACACTGCAGGAGATG)
		Reverse (TGAGGAATCACCCACAGG)
*nkaα3*	PCR	Forward (TGACAACCAAATCCATGAG)
		Reverse (AGGTGCTGAAGAATCATACT)
	5’ RACE-PCR	TTGTTGGTGGAGTTGAAGGGAATCTCAG
	3’ RACE-PCR	TTCGATACCGATGACATCAACTTCCAG
	qPCR	Forward (ATCCTGAAGAGGGACGTG)
		Reverse (GTGCATGAGACAGAAGACTC)

### Rapid Amplification of cDNA Ends (RACE)-PCR

Total RNA (1 μg) isolated from the muscle, main EO, Hunter’s EO or Sach’s EO of *E*. *electricus* was reverse transcribed into 5’-RACE-Ready cDNA and 3’RACE-Ready cDNA using SMARTer RACE cDNA Amplification kit (Clontech Laboratories, Mountain View, CA, USA). RACE-PCR was performed using the Advantage 2 PCR kit (Clontech Laboratories) to generate the 5’ and 3’ cDNA fragments. The cycling conditions comprised 30 cycles of 94°C for 30 s, 65°C for 30 s, and 72°C for 4 min. RACE-PCR products were separated using gel electrophoresis, purified and sequenced. Multiple sequencing was performed in both directions to obtain the full-length cDNA. Sequence assembly and analysis were performed using Bioedit v7.1.3 [27].

### Deduced amino acid sequences and phenogramic analysis

The Nkaα amino acid sequences were translated from the respective *nkaα* nucleotide sequences using ExPASy Proteomic server (http://web.expasy.org/translate/). The deduced amino acid sequence was aligned and compared with selected Nkaα1, α2 and α3 from various animal species using BioEdit. The transmembrane domains were predicted using MEMSAT3 and MEMSAT-SVM provided by PSIPRED protein structure prediction server (http://bioinf.cs.ucl.ac.uk/psipred/) [28].

Furthermore, these sequences were aligned using ClustalX2 and phenogramic analyses were performed using neighbor-joining method and 100 bootstrap replicates with Phylip v3.6 [29].

Amino acid sequences of Nkaα1, Nkaα2 and Nkaα3 from other animals were obtained from Genbank or UniProtKB/TrEMBL with the following accession numbers: *Anabas testudineus* Nkaα1a (AFK29492.1), Nkaα1b (AFK29493.1) and Nkaα1c (AFK29494.1); *Oncorhynchus masou* Nkaα1a (BAJ13363) and Nkaα1b (BAJ13362); *Oncorhynchus mykiss* Nkaα1a (NP_001117933), Nkaα1b (NP_001117932), Nkaα1c (NP_001117931), Nkaα2 (NP_001117930) and Nkaα3 (NP_001118102); *Saccoglossus kowalevskii* Nka (XP_002737354); *Squalus acanthias* Nkaα (CAG77578) and *Torpedo californica* Nkaα (P05025.1).

### qPCR

There are two types of quantification methods for qPCR [30]. Relative quantitation involves the comparison of the targeted gene with a reference gene, and produce only fold-change data, which do not allow the interpretation of which isoform being the predominant one expressed in a certain tissue or under a certain condition. For absolute quantification, the precise amount of the template used for the standard curve is known, and therefore results of the targeted gene can be expressed as absolute numbers of copies of transcripts. Although absolute quantification provides more information, it is considered to be more labor-intensive than relative quantification, and is not commonly adopted because of the necessity to create reliable standards for quantification and to include these standards in every PCR run. Since it is essential to compare the mRNA expression levels of various *nkaα* isoforms among the SM and the three EOs of *E*. *electricus*, the method of absolute quantification with reference to 4 standard curves was adopted in this study.

RNA (4 μg) from various tissues samples of *E*. *electricus* were extracted as mentioned above and reverse-transcribed using random hexamer primers with RevertAid first strand cDNA synthesis kit (Thermo Fisher Scientific Inc.). qPCR was performed in triplicates using a StepOnePlus Real-Time PCR System (Applied Biosystems). The mRNA expression of the four *nkaα*-subunits in the SM, main EO, Hunter’s EO and Sach’s EO of *E*. *electricus* was determined using specific qPCR primers (Table 1).

In order to determine the absolute quantity of the each of the *nkaα-*subunit transcripts in a qPCR reaction, efforts were made to produce a pure amplicon (standard) of a defined region of each *nkaα* isoform from *E*. *electricus* following the method of Gerwick et al. [31]. PCR was performed with the qPCR primers (Table 1) and cDNA as a template in a final volume of 25 μl with the following cycling conditions: initial denaturation 95°C for 3 min, followed by 35 cycles of 95°C for 30 s, 60°C for 30 s and 72°C for 30 s and 1 cycle of final extension of 72°C for 10 min. The PCR product was separated in a 2% agarose gel then excised and purified using Promega Wizard SV gel and PCR cleanup system. The *nka α*-subunit nucleotide fragment in the purified product was cloned using pGEM-T Easy vector. The presence of the insert in the recombinant clones was confirmed by sequencing. The cloned circular plasmid was quantified using a spectrophotometer.

The standard cDNA (template) was serially diluted (from 10^6^ to 10^2^ specific copies per 2 μl). The PCR reactions contained 5 μl of KAPA SYBR FAST Master Mix (2X) ABI Prism (Kapa Biosystems, Woburn, MA, USA), 0.3 μmol l^-1^ of forward and reverse primers each (Table 1) and 1 ng of sample cDNA or various quantities of standard in a total volume of 10 μl. Cycling conditions were 95°C for 20 s (1 cycle), followed by 40 cycles of 95°C for 3 s and 60°C for 30 s. Data (Ct values) were collected at each elongation step. A melt curve analysis was performed after each run by increasing the temperature from 60°C to 95°C in 0.3°C increments to confirm the presence of a single product only. The PCR products obtained were also separated in a 2% agarose gel to verify the presence of a single band. A standard curve was obtained from plotting threshold cycle (C_t_) on the Y-axis and the natural log of concentration (copies μl^-1^) on the X-axis. The C_t_ slope, PCR efficiency, Y-intercept and correlation coefficient (r^2^) were calculated using the default setting of StepOne Software v2.1. Diluted standards were stored at -20°C. The PCR amplification efficiencies for *nkaα1c1*, *nkaα1c2*, *nkaα2* and *nkaα3* were 96.5%, 93.1%, 90.7% and 91.8%, respectively. The quantity of transcript in a sample was determined from the linear regression line derived from the standard curve and expressed as copy number per ng cDNA [31].

The specificity of each pair of qPCR primers was verified by PCR using four different plasmid clones containing fragments of *nkaα1c1*, *nkaα1c2*, *nkaα2* or *nkaα3* as templates. The identities of these plasmid clones had been verified through cloning and sequencing previously (see section above). The specificity of each pair of primers was demonstrated by the presence of a single band using the plasmid clones of the targeted *nkaα* isoform as the template and the absence of detectable band using the plasmid clones of the other isoform. Furthermore, for each pair of primers, the C_t_ values obtained using plasmid clones of the targeted *nkaα* fell between 14 and 32, but no detectable C_t_ values (i.e., undetermined) were obtained using the other plasmid clones.

### SDS-Page electrophoresis and western blotting

Tissues samples from SM or EOs were homogenized three times in five volumes (w/v) of ice cold buffer containing 50 mmol l^-1^ Tris HCl, (pH 7.4), 1 mmol l^-1^ EDTA, 150 mmol l^-1^ NaCl, 1 mmol l^-1^ NaF, 1 mmol l^-1^ Na_3_VO_4_, 1% NP-40, 1% sodium deoxycholate, 1 mmol l^-1^ PMSF, and 1x HALT protease inhibitor cocktail (Thermo Fisher Scientific Inc.) at 24,000 rpm for 20 s each with 10 s intervals using the Polytron PT 1300D homogenizer (Kinematica AG, Lucerne, Switzerland). The homogenate was centrifuged at 10,000 ×g for 20 min at 4C. The protein concentration in the supernatant obtained was determined according to the method of Bradford [35] and adjusted to 5 μg μl^-1^ with Laemmli buffer [32]. Samples were heated at 70C for 15 min, and then kept at -80°C until analysis. The protein load for the SM, the main EO, Hunter’s EO and Sach’s EO were 100 μg each.

Proteins were separated by SDS-PAGE (8% acrylamide for resolving gel, 4% acrylamide for stacking gel) under conditions as described by Laemmli [32] using a vertical mini-slab apparatus (Bio-Rad Laboratories, Hercules, CA, USA). Preliminary experiments indicated that the protein abundances of Nkaα in the EOs of adult fish were higher than those in the EOs of juvenile fish. Therefore, 100 μg and 20 μg of protein from the SM/EOs of juvenile and adult fish, respectively, were loaded for SDS-PAGE. After SDS-PAGE, separated proteins were electrophoretically transferred onto PVDF membranes using a transfer apparatus (Bio-Rad Laboratories). After transfer, membranes were blocked with 10% skim milk in TTBS (0.05% Tween 20 in Tris-buffered saline: 20 mmol l^-1^ Tris-HCl; 500 mmol l^-1^ NaCl, pH 7.6) for 1 h before being incubated overnight at 4C with the anti-NKAα antibody, α5 (1:800 dilution). α5 was developed by Douglas M. Farmbrough (Johns Hopkins University, MD, USA) under the auspices of NICHD and maintained by The University of Iowa, Department of Biological Sciences, Iowa City, IA52242, USA, and is known to react pan-specifically with Nkaα isoforms in fish and other animals [33,34]. The α5 antibody was diluted in 1% bovine serum albumin BSA in TTBS. The membranes were then incubated in goat anti-mouse horseradish peroxidase-conjugated secondary antibodies (1:10,000; Santa Cruz Biotechnology Inc.) for 1 h at room temperature. Bands were visualized by chemiluminescence (Western Lightning, PerkinElmer Life Sciences, Boston, MA, USA) using X-ray film (Thermo Fisher Scientific) and were processed by a Kodak X-Omat 3000 RA processor (Kodak, Rochester, NY, USA). The films were scanned using CanonScan 4400F flatbed scanner in TIFF format at 300 dpi resolution. Densitometric quantification of band intensities were performed using ImageJ (version 1.40, NIH), calibrated with a 37 step reflection scanner scale (#R3705–1C; StoufferGraphicArts, SouthBend, IN, USA) calibrated with a calibrated 37 step reflection scanner scale (1” x 8”; Stouffer #R3705–1C).

Usually, a reference protein, e.g. β-actin or glyceraldehyde 3-phosphate dehydrogenase, is included in western blotting to confirm that the experimental condition tested leads to changes in the abundance of the targeted protein, but not the reference protein, in the same tissue/organ as compare to the control condition. However, in this study, we aimed to compare the protein abundances of Nkaα among 4 different tissue/organs in *E*. *electricus* whereby no change in experimental conditions, and hence no control, were involved. Preliminary studies confirmed that the protein abundances of β-actin and glyceraldehyde 3-phosphate dehydrogenase differed significantly among the SM and the three EOs of *E*. *electricus*, indicating that they were not suitable to act as reference proteins. Therefore, for valid comparisons, western blotting results were presented as relative protein abundance of Nkaα per 100 μg protein for juvenile fish or per 20 μg protein for adult fish.

### Determination of Nka activity and kinetic properties

Tissues samples of SM or EOs were thawed on ice and homogenized for 2 s at 7,000 rpm. The homogenate was then centrifuged at 2000 ×g for 7 min at 4°C to obtain the pellet. The pellet was re-suspended in 1 ml of homogenizing buffer containing 0.1 mol l^-1^ imidazole-HCl (pH 7.2), 0.3 mol l^-1^ sucrose, and 1 g l^-1^ of sodium deoxycholate (without EDTA, which interfered with the subsequent phosphate analysis), and homogenized twice at 13,500 rpm for 10 s each with an interval of 10 s. The homogenized sample was centrifuged for 6 min at 2,000 ×g and 4°C. The supernatant obtained was assayed for Nka activity on the same day. The protein content of the sample was determined according to the method of Bradford [35]. The quantities of protein used per assay were approximately 0.1 mg and 0.5 mg for EOs and SM, respectively.

The optimized reaction mixture for the determination of Nka activity (*V*_sat_) contained 0.05 ml sample, 30 mmol l^-1^ imidazole-HCl buffer (pH 7.2), 120 mmol l^-1^ NaCl, 20 mmol l^-1^ KCl, 5 mmol l^-1^ MgCl_2_ and 3.5 mmol l^-1^ ATP, with or without 2 mmol l^-1^ ouabain in a total volume of 1 ml. The compositions of Na^+^ and K^+^ (or NH_4_^+^ in replacement of K^+^) were varied in order to obtain the *K*_*m*_ for various substrates (Na^+^, K^+^ and NH_4_^+^) and the corresponding *V*_max_. The reaction mixture without ATP was pre-incubated at 25°C for 10 min and the reaction was initiated by the addition of 0.05 ml of ATP. After 40 min of incubation at 25°C, the reaction was terminated by the addition of 0.05 ml of ice-cold 100% trichloroacetic acid. The mixture was centrifuged at 12,000 ×*g* for 2 min at 4°C. Preliminary results indicate that the Nka activity increased linearly with time up to at least 45 min under all conditions. The amount of inorganic phosphate (Pi) released from ATP during the incubation period represented the activity of Nka. An aliquot (0.4 ml) of the supernatant was diluted with 4 volumes of 0.1 mol l^-1^ sodium acetate for Pi assay. To this diluted aliquot, 0.2 ml of 1% ascorbic acid and 0.2 ml of 1% ammonium molybdate in 0.05 mol l^-1^ H_2_SO_4_ were added. The solution was incubated for 30 min at 30°C and the absorbance was determined at 700 nm using a Shimadzu (Kyoto, Japan) UV160 UV-VIS spectrophotometer, and the Pi concentration calculated with reference to a standard made from K_2_HPO_4_ and assayed in the presence of trichloroacetic acid and sodium acetate. The Nka activity was calculated as a difference of activities assayed in the presence and absence of ouabain, and expressed as μmol Pi releasí·min^-1^·g^-1^ tissue wet mass. Of note, the deoxycholate-extractable protein content (mg protein per g wet mass tissue) varied between the SM and the three EOs. Hence, it was essential to present the results as mass-specific activities instead of protein-specific activities for valid comparisons.

To obtain *K*_*m*_ and *V*_max_ for Na^+^, effects of different concentrations of Na^+^ (2.5, 5, 10, 20, 40, 60 and 120 mmol l^-1^) on Nka activity were determined in the presence of 20 mmol l^-1^ K^+^ for all samples. To obtain *K*_*m*_ and *V*_max_ for K^+^ or NH_4_^+^, effects of varying K^+^ or NH_4_^+^ concentrations (0.25, 0.5, 1, 2, 4, 8, 10 and 20 mmol l^-1^) on the Na^+^/K^+^-ATPase or Na^+^/NH_4_^+^-ATPase activity were determined at 120 mmol l^-1^ Na^+^. The *K*_*m*_ and *V*_max_ values were obtained from the Woolf—Augustinsson plot. Two *V*_max_ values were generated for each samples based on varied Na^+^, or K^+^ concentrations.

### Statistical analysis

Due to the small sampling size of the adult fish, results reported herein were obtained mainly from the juvenile fish unless stated otherwise. Results were presented as means ± standard errors of means (S.E.M.). Statistical analyses were performed using SPSS version 21 (IBM Corporation, Armonk, NY, USA). Homogeneity of variance was checked using Levene’s Test. For results on western blotting and Nka kinetic properties, differences between means were tested using one-way analysis of variance (ANOVA) followed by multiple comparisons of means by Dunnett’s T3 (for unequal variance) or by Tukey’s test (for equal variance). Except for qPCR results presented on *nkaα2* and *nkaα3* for adult eels, the rest of the qPCR results showed equal variance and were therefore analysed by ANOVA followed with Duncan’s post-hoc test. For qPCR results presented on *nkaα2* and *nkaα3* for adult eels, ANOVA and Duncan’s test were applied after square root transformation. Differences were regarded as statistically significant at P<0.05.

## Results

### Nucleotide and amino acid sequences of *nkaα*/Nkaα isoforms

**Fig 1:**
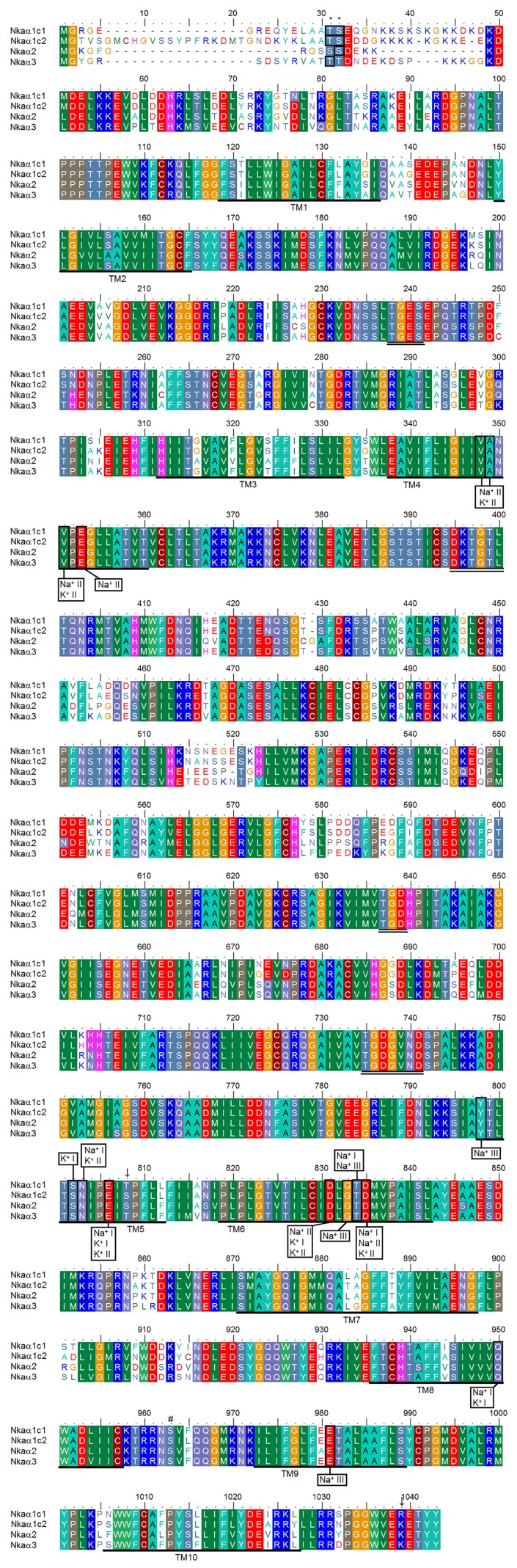
Molecular characterization of Na^+^/K^+^-ATPase α-subunit (Nkaα) isoforms of *Electrophorus electricus*. A multiple amino acid alignment of Nkaα1c1, Nkaα1c2, Nkaα2 and Nkaα3 of *E*. *electricus*. Identical or strongly similar amino acids are indicated by shaded residues. The 10 predicted transmembrane regions (TM1–TM10) are underlined. Vertical boxes represent coordinating residues for Na^+^ or K^+^ binding. The conserved regions containing the TGES, DKTGTL, TGD and TGDGVND sequence motifs are double underlined. The hash marks denote the amino acid residues phosphorylated by PKA. The asterisks denote the amino acid residues phosphorylated by PKC. Substitution of amino acids is indicated by an arrow. The transmembrane domains of Nkaα1c1, Nkaα1c2, Nkaα2 and Nkaα3 of *E*. *electricus* were predicted using MEMSAT3 and MEMSAT-SVM provided by PSIPRED protein structure prediction server.

Coding cDNA sequences of *nkaα1c1* (Genbank accession number: KM282053), *nkaα1c2* (KM282054) *nkaα2* (KM282055) and *nkaα3* (KM282056) were cloned from the SM and EOs of *E*. *electricus*. The complete coding sequence of *nkaα1c1* consisted of 3081 bp, which encoded 1026 amino acids with an estimated molecular mass of 113.1 kDa (Fig. 1). On the other hand, the complete coding cDNA sequence of *nkaα1c2* comprised 3123 bp, encoding 1040 amino acids with an estimated molecular mass of 114.4 kDa (Fig. 1). For *nkaα2*, the coding sequence consisted of 3030 bp, encoding 1009 amino acids with an estimated molecular mass of 110.0 kDa (Fig. 1). For *nkaα3*, the coding sequence consisted of 3069 bp, encoding 1022 amino acids with an estimated molecular mass of 112.7 kDa (Fig. 1).

**Fig 2:**
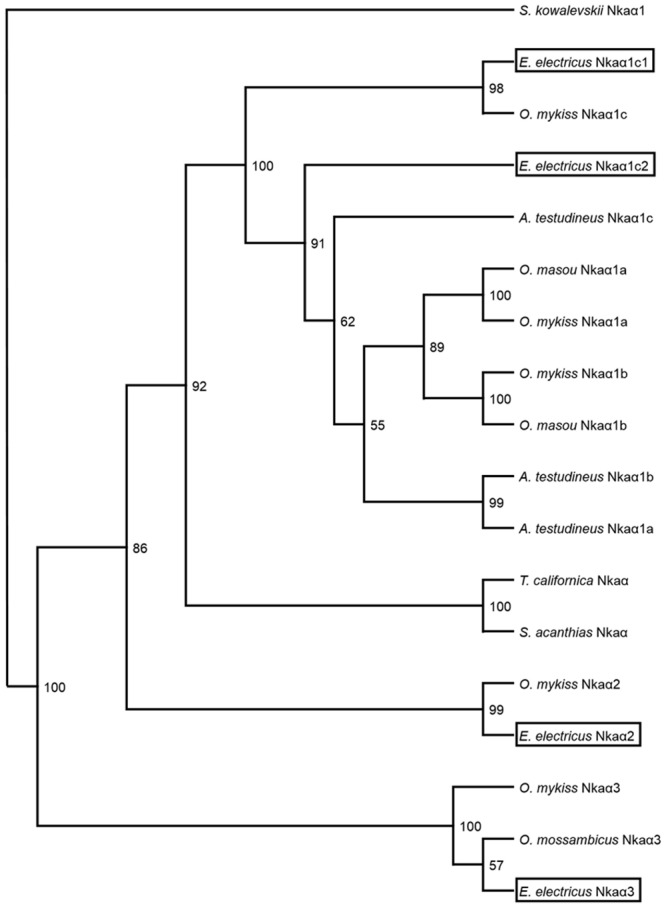
A phenogramic analysis of Na^+^/K^+^-ATPase α-subunit (Nkaα) isoforms of *Electrophorus electricus*. A phenogram illustrating the relationship among Nkaα1c1, Nkaα1c2, Nkaα2 and Nkaα3 of *E*. *electricus* and Nkaα isoforms of selected animals with Nkaα1 of *Saccoglossus kowalevskii* as the outgroup. The number at each branch represents the bootstrap value (max = 100).

The identities of the deduced amino acid sequences of the four isoforms of Nkaα from *E*. *electricus* were first obtained through a comparison of percentage similarities with various Nkaα isoforms from teleost species available in GenBank (Table 2). As we had obtained two Nkaα1 isoforms from *E*. *electricus*, *A*. *testudineus*, *O*. *mykiss* and *O*. *masou* were chosen for comparison because subforms of their Nkaα1 have been identified. Results indicated the two forms of Nkaα1 from *E*. *electricus* were *nkaα1c1* and *nkaα1c2* (Table 2). Subsequently, a phenogramic analysis was performed (Fig. 2), which demonstrated that the two Nkaα1c isoforms (Nkaα1c1 and Nkaα1c2) were indeed grouped together under the same clade with Nkaα1 of other teleost fishes, with Nkaα2 and Nkaα3 being grouped separately (Fig. 2). The Nkaα2 sequence obtained from this study was identical to the Nkaα sequence (AF356351) reported by Kaya et al. [26].

**Table 2:** Percentage similarity between the amino acid sequences of Na^+^/K^+^-ATPase α1c1 (Nkaα1c1), Nkaα1c2, Nkaα2 and Nkaα3 from *Electrophorus electricus* and Nkaα sequences from other teleosts obtained from GenBank.

	Fish species	Isoform	*Electrophorus electricus*			
			Nkaα1c1	Nkaα1c2	Nkaα2	Nkaα3
Nkaα1	*Oncorhynchus mykiss*	Nkaα1c	**91.7**	86.8	80.6	83.3
	*Anabas testudineus*	Nkaα1c	**89.0**	**88.6**	80.9	82.6
	*Oncorhynchus masou*	Nkaα1b	88.4	**88.6**	79.9	82.1
	*Oncorhynchus mykiss*	Nkaα1b	88.3	88.4	80.1	82.1
	*Anabas testudineus*	Nkaα1b	85.7	83.7	78.6	79.7
	*Oncorhynchus mykiss*	Nkaα1a	82.1	81.3	74.7	76.3
	*Oncorhynchus masou*	Nkaα1a	81.8	81.3	74.5	76.3
	*Anabas testudineus*	Nkaα1a	80.0	78.3	73.7	76.9
Nkaα2	*Fundulus heteroclitus*	Nkaα2	82.8	80.6	**85.2**	81.5
	*Oncorhynchus mykiss*	Nkaα2	82.5	79.6	**84.2**	81.4
Nkaα3	*Carassius auratus*	Nkaα3	84.9	82.0	80.5	**95.3**
	*Oreochromis mossambicus*	Nkaα3	82.2	79.2	80.0	**90.4**
	*Oncorhynchus mykiss*	Nkaα3	82.5	79.3	79.1	89.4
	*Trematomus bernacchii*	Nkaα3	82.5	81.2	79.4	88.0

Similarity indices were obtained using the BioEdit software.

There were ten predicted transmembrane regions (TM1–TM10) in all four isoforms with lengths of 15 to 27 amino acids (Fig. 1). Highly conserved motifs were identified including the threonine-glycine-glutamic acid-serine (TGES) motif, the aspartic acid-lysine-threonine-glycine-threonine (DKTGT) motif, the threonine-glycine-aspartic acid (TGD) motif and the threonine-glycine-aspartic acid-glycine-valine-asparagine-aspartic acid (TGDGVND) motif. The sites of phosphorylation by protein kinase A (PKA) and protein kinase C (PKC) as well as the binding sites for Na^+^ and K^+^ were also identified and labelled (Fig. 1). Two cases of amino acid substitution were also indicated by arrows.

### Absolute quantification of mRNA expression levels

**Fig 3:**
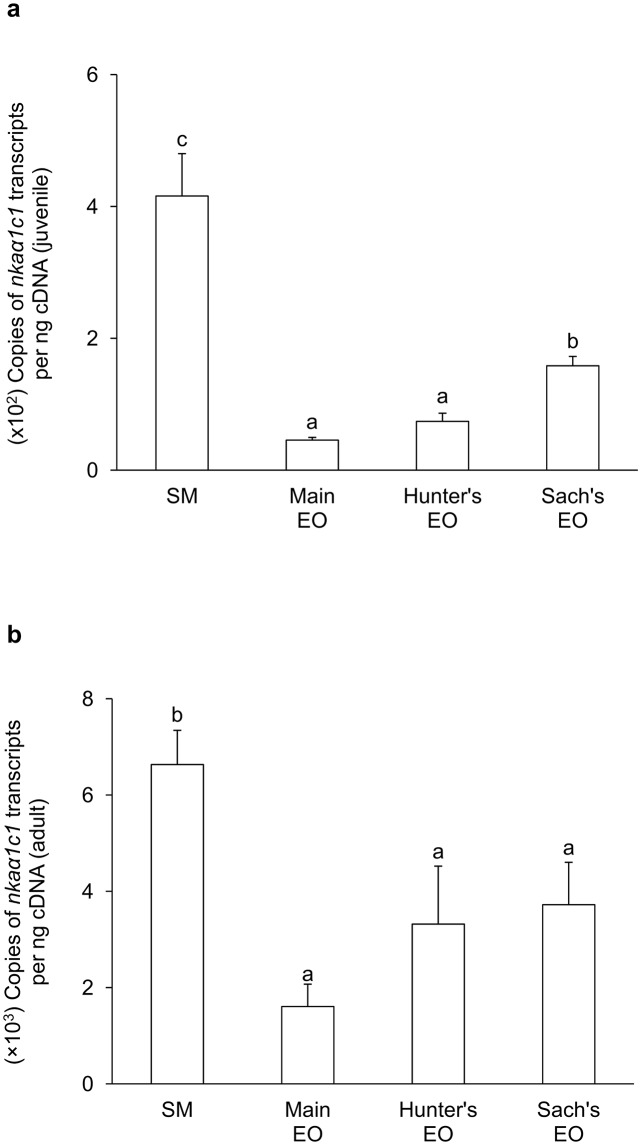
mRNA expression levels of *Na* ^+^ */K* ^+^ *-ATPase α1c1* (*nkaα1c1*) in *Electrophorus electricus*. Absolute quantification (copies of transcript per ng of cDNA) of mRNA expression levels of *nkaα1c1* in the skeletal muscle (SM), the main electric organ (EO), the Hunter’s EO and the Sach’s EO of (a) juvenile (*N* = 5) and (b) adult (*N* = 3) *E*. *electricus* kept in freshwater. Results represent mean + S.E.M. Means not sharing the same letter are significantly different (P<0.05).

**Fig 4:**
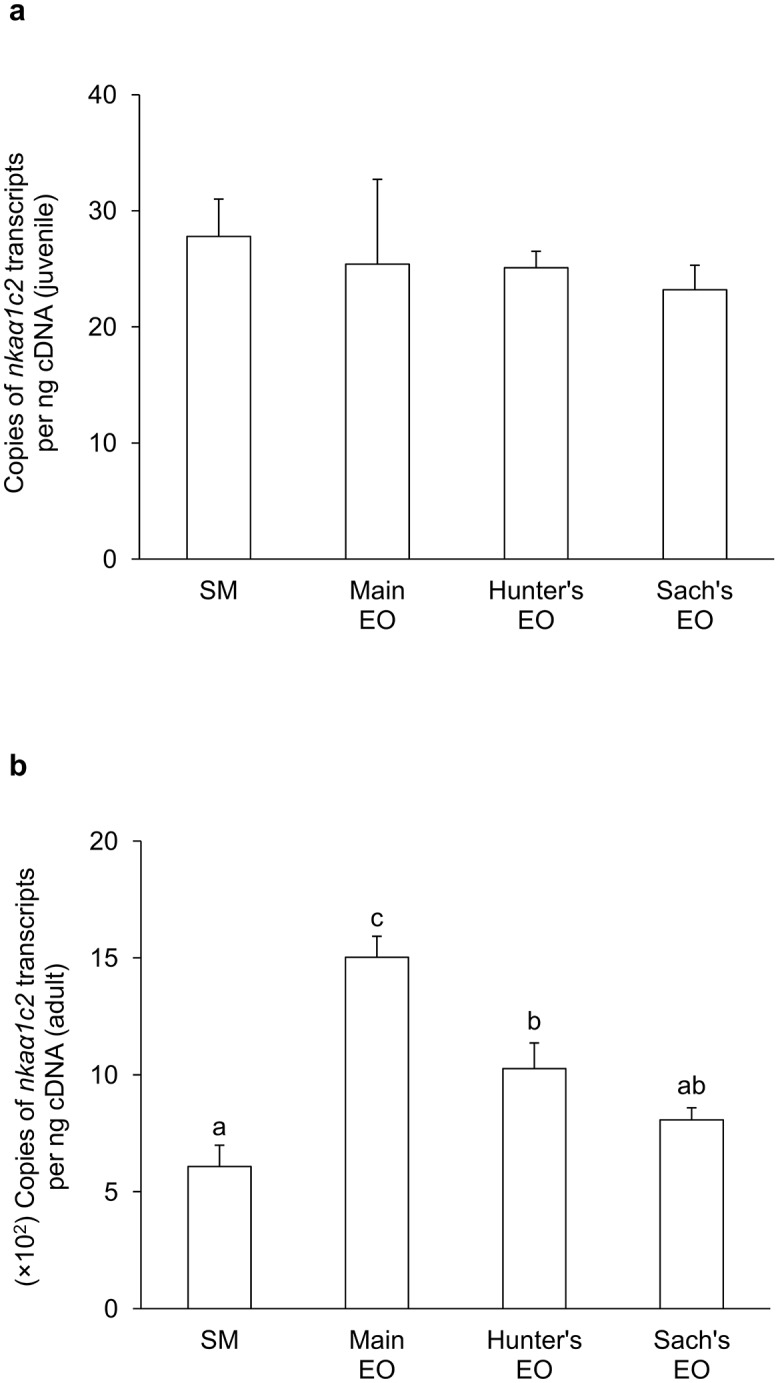
mRNA expression levels of *Na* ^+^ */K* ^+^ *-ATPase α1c2* (*nkaα1c2*) in *Electrophorus electricus*. Absolute quantification (copies of transcript per ng of cDNA) of mRNA expression levels of *nkaα1c2* in the skeletal muscle (SM), the main electric organ (EO), the Hunter’s EO and the Sach’s EO of (a) juvenile (*N* = 5) and (b) adult (*N* = 3) *E*. *electricus* kept in freshwater. Results represent mean + S.E.M. Means not sharing the same letter are significantly different (P<0.05).

**Fig 5:**
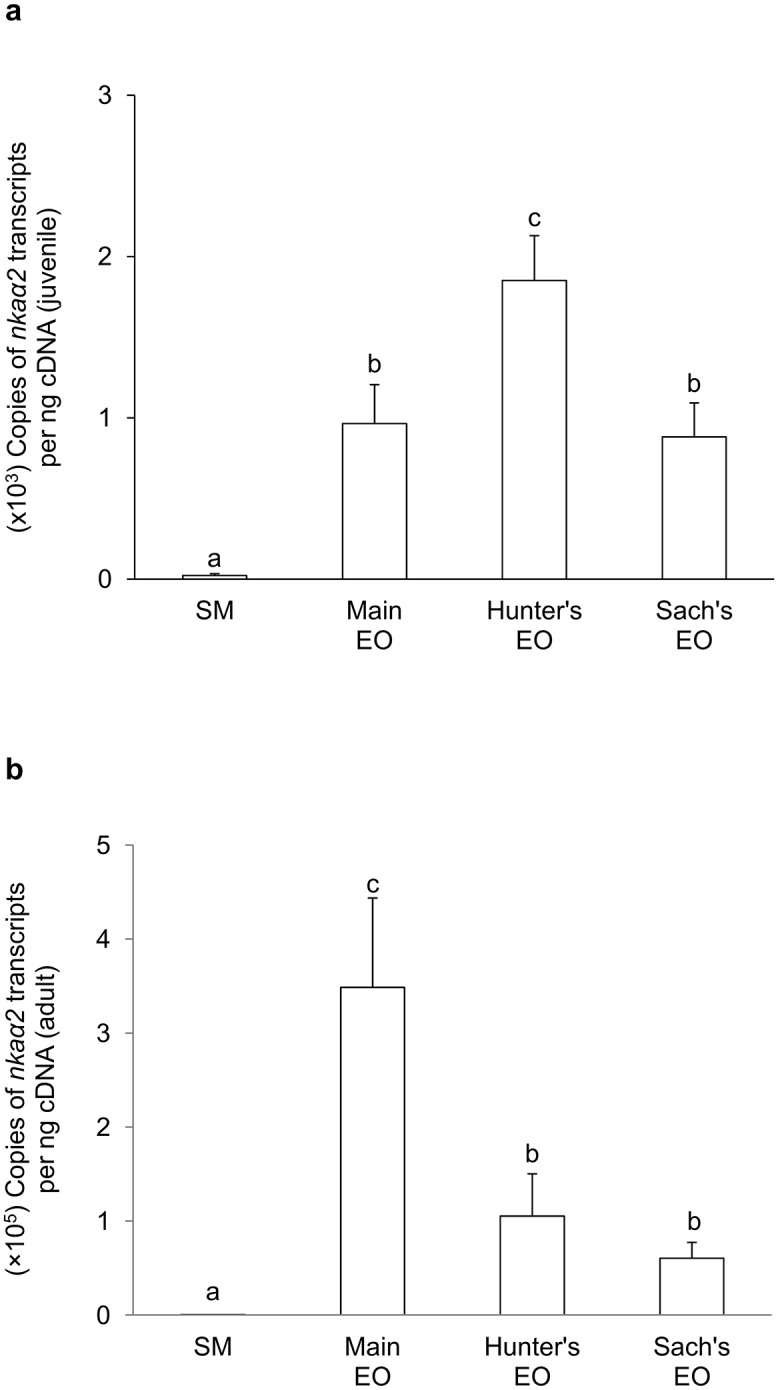
mRNA expression levels of *Na* ^+^ */K* ^+^ *-ATPase α2* (*nkaα2*) in *Electrophorus electricus*. Absolute quantification (copies of transcript per ng of cDNA) of mRNA expression levels of *nkaα2* in the skeletal muscle (SM), the main electric organ (EO), the Hunter’s EO and the Sach’s EO of (a) juvenile (*N* = 5) and (b) adult (*N* = 3) *E*. *electricus* kept in freshwater. Results represent mean + S.E.M. Means not sharing the same letter are significantly different (P<0.05).

**Fig 6:**
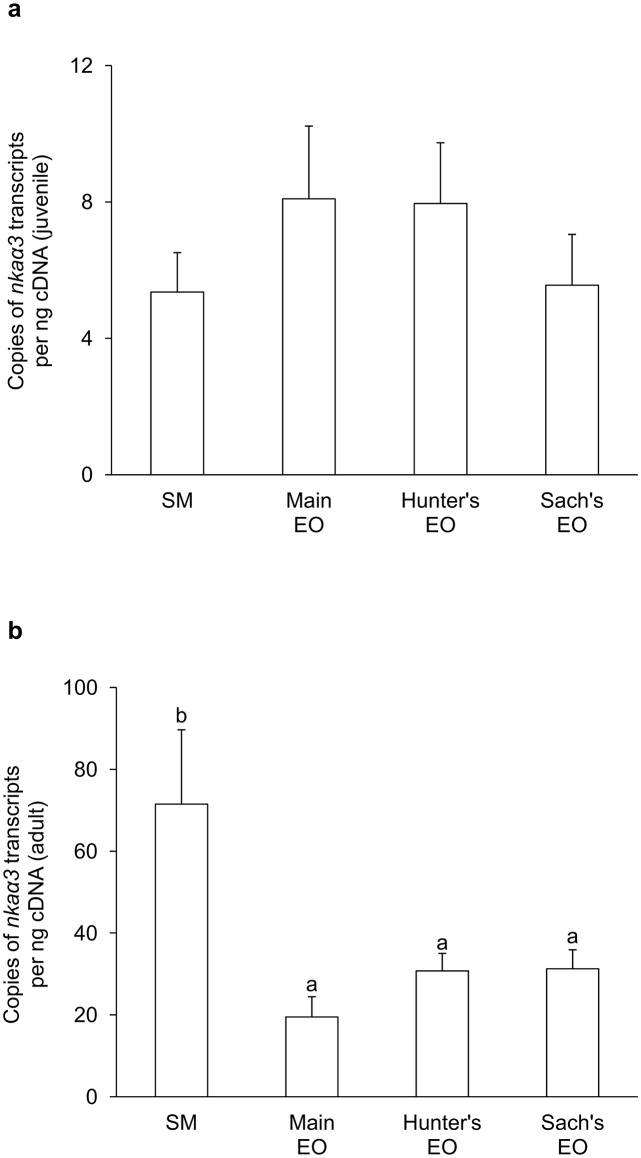
mRNA expression levels of *Na* ^+^ */K* ^+^ *-ATPase α3* (*nkaα3*) in *Electrophorus electricus*. Absolute quantification (copies of transcript per ng of cDNA) of mRNA expression levels of *nkaα3* in the skeletal muscle (SM), the main electric organ (EO), the Hunter’s EO and the Sach’s EO of (a) juvenile (*N* = 5) and (b) adult (*N* = 3) *E*. *electricus* kept in freshwater. Results represent mean + S.E.M. Means not sharing the same letter are significantly different (P<0.05).

For juvenile *E*. *electricus*, the mRNA expression level of *nkaα1c1* in the SM (416 copies per ng cDNA) was significantly higher than those in the three EOs (9-fold of the main EO; 6-fold of the Hunter’s EO and 3-fold of the Sach’s EO; Fig. 3a). Hence, *nkaα1c1* could be regarded as a SM-predominant isoform. The mRNA expression level of *nkaα1c2* (~25 copies per ng cDNA) was substantially lower than that of *nkaα1c1* in SM, and there were no significant differences in *nkaα1c2* mRNA expression levels among the SM and the three EOs (Fig. 4a). On the other hand, the mRNA expression levels of *nkaα2* in the three EOs were significantly higher than that in the SM (Fig. 5a), indicating that it was an EO-predominant form. Furthermore, the mRNA expression level of *nkaα2* in the Hunter’s EO was significantly higher than those in the main EO and the Sach’s EO. The mRNA expression level of *nkaα3* was the lowest among the four *nkaα* isoforms, and it had comparable expression levels among the SM and the three EOs (Fig. 6a).

In general, the mRNA expression levels of all four *nkaα* isoforms in the SM and EOs of adult *E*. *electricus*, (Figs. 3b–6b) were higher than those of the juvenile fish (Figs. 3a–6a). For adult fish, the mRNA expression level of *nkaα1c1* in the SM (~6500 copies per ng cDNA) was significantly higher than those in the three EOs (4-fold of the main EO; 2-fold of the Hunter’s EO and Sach’s EO; Fig. 3b), confirming that it was a SM-predominant isoform. The mRNA expression level of *nkaα1c2* (~600 copies per ng cDNA) in the SM was only 1/10 that of *nkaα1c1*, and it was comparable to that of *nkaα1c2* in the Sach’s EO but significantly lower than those in the main EO and the Hunter’s EO (Fig. 4b). Results obtained from adult *E*. *electricus* confirmed that *nkaα2* was EO-predominant, but unlike juvenile fish, its expression level in the main EO (~340000 copies per ng cDNA) was significantly higher than those in the Hunter’s EO and the Sach’s EO (Fig. 5b). Like juvenile fish, the mRNA expression level of *nkaα3* (~70 copies per ng cDNA) was the lowest among the four *nkaα* isoforms in the SM of adult *E*. *electricus*, and its expression level in the SM was significantly higher than those in the three EOs (Fig. 6b).

### Pan-Nkaα protein abundance

**Fig 7:**
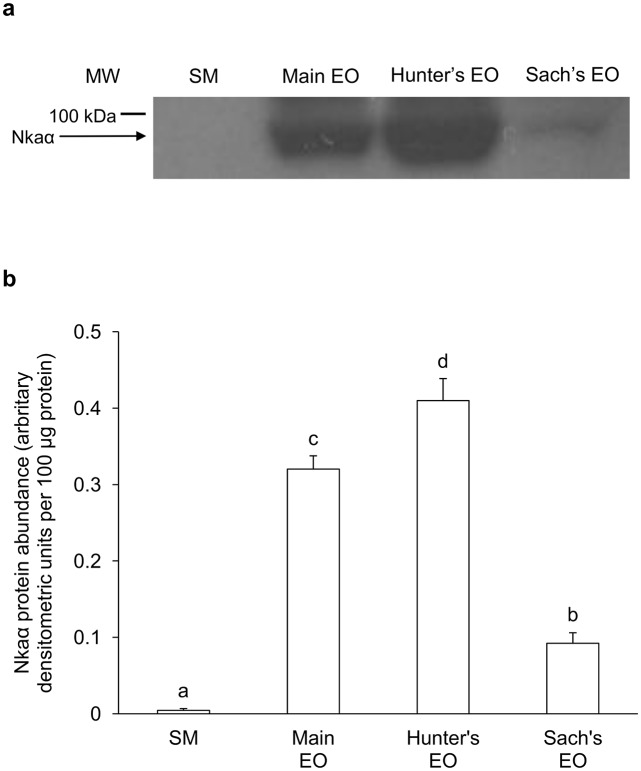
Western blotting results of Na^+^/K^+^-ATPase α-subunit (Nkaα) in juvenile *Electrophorus electricus*. Protein abundance of Nkaα in the skeletal muscle (SM), the main electric organ (EO), the Hunter’s EO and the Sach’s EO of juvenile *E*. *electricus* kept in freshwater. (a) An example of the immunoblot of Nkaα. (b) The protein abundance of Nkaα expressed as arbitrary densitometric units per 100 μg protein. Results represent mean + S.E.M (*N* = 3). Means not sharing the same letter are significantly different among the SM and the three EOs (P<0.05).

**Fig 8:**
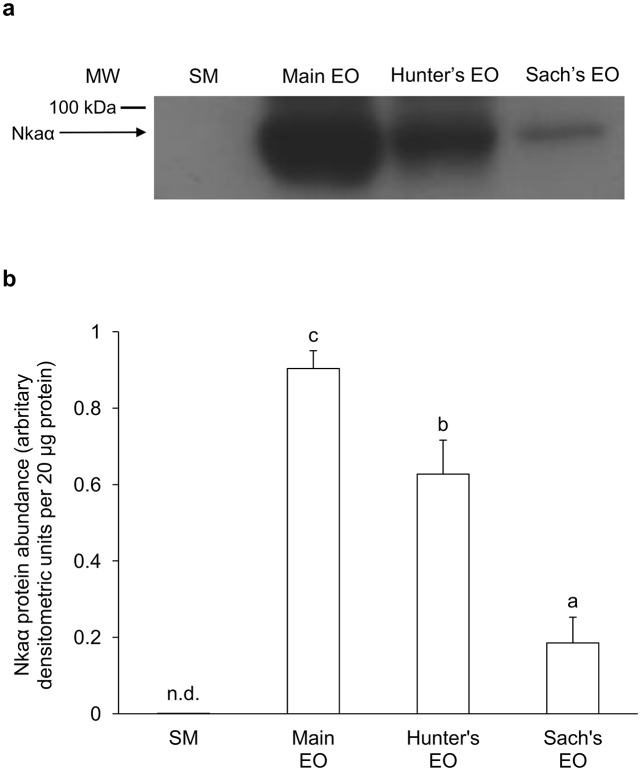
Western blotting results of Na^+^/K^+^-ATPase α-subunit (Nkaα) in adult *Electrophorus electricus*. Protein abundance of Nkaα in the skeletal muscle (SM), the main electric organ (EO), the Hunter’s EO and the Sach’s EO of adult *E*. *electricus* kept in freshwater. (a) An example of the immunoblot of Nkaα. (b) The protein abundance of Nkaα expressed as arbitrary densitometric units per 20 μg protein. Results represent mean + S.E.M (*N* = 3). Means not sharing the same letter are significantly different among the three EOs (P<0.05). Nkaα was not detectable (n.d.) in the SM.

Based on the pan-isoform-specific anti-NKA α5 antibody, the Hunter’s EO of juvenile *E*. *electricus* had the highest protein abundance of Nkaα, followed by the main EO and the Sach’s EO, but Nkaα was barely detectable in the SM (Fig. 7). As for adult fish, Nkaα was undetectable in the SM, but expressed strongly in the main EO and the Hunter’s EO, with the former having significantly higher Nkaα protein abundance than the latter. Nkaα was only weakly expressed in the Sach’s organ of adult fish (Fig. 8). In general, the protein abundance Nkaα in the EOs of the adult fish (Fig. 8) were much higher than those in the juvenile fish (Fig. 7).

### Activities and kinetic properties of Nka

The *V*_sat_ (μmol Pi·min^-1^·g^-1^ tissue; *N* = 4) of Nka for the SM (0.44 ± 0.23) was significantly lower than those of the main EO (5.05 ± 1.28), the Hunter’s EO (3.03 ± 0.31) and the Sach’s EO (2.31 ± 0.42) of juvenile *E*. *electricus*. Due to the very low activity of Nka in the SM, it was difficult to accurately obtain the *K*_*m*_ values for Na^+^ and K^+^. Hence, we focused on the kinetic properties of Nka from the three EOs. In corroboration with the *V*_sat_, the highest *V*_max_ was obtained, based on varying Na^+^ or K^+^ concentrations, for the Nka from the main EO, and Nka from the Sach’s EO had the lowest *V*_max_ value (Table 3). The *K*_*m*_ of Na^+^ for the Nka from the main EO was significantly lower than that from the Sach’s EO. Additionally, the *K*_*m*_ of K^+^ for Nka from the main EO was significantly lower than those from the Hunter’s EO and the Sach’s EO (Table 3).

**Table 3:** *V*_max_ (μmol Pi released·min^-1^·g^-1^ tissue; *N* = 4) and *K*_*m*_ (mmol l^-1^; *N* = 4) values of Na^+^/K^+^-ATPase (Nka) from the main electric organ (EO), the Hunter’s EO and the Sach’s EO of juvenile *Electrophorus electricus*.

Nka	Main EO	Hunter’s EO	Sach’s EO
*V*_max_ (based on Na^+^)	6.60 ± 1.46^b^	4.06 ± 0.45^ab^	2.85 ± 0.51^a^
*K*_*m*_ [Na^+^]	7.47 ± 1.29^a^	18.14 ± 5.94^ab^	24.20 ± 2.44^b^
*V*_max_ (based on K^+^)	5.86 ± 1.02^b^	3.67 ± 0.45^ab^	2.49 ± 0.44^a^
*K*_*m*_ [K^+^]	0.46 ± 0.14^a^	1.47 ±0.30^b^	1.20 ± 0.08^b^

Results represent mean ± S.E.M. Means not sharing the same letter are significantly different (P<0.05).

## Discussion

In EOs, electrocytes are activated by motor neurons that form synapses with the innervated membrane [36]. Neurons release the neurotransmitter acetylcholine which induces the opening of the voltage-gated Na^+^ channels when bound to the acetylcholine receptors on the extracellular side of the innervated membrane. These voltage-gated Na^+^ channels, which are located primarily along the innervated membrane [37], allows for an influx of Na^+^ down the electrochemical gradient causing a momentary localized depolarization of the innervated membrane [15,18]. Acetylcholinesterase then acts on the neurotransmitter and removes them from the acetylcholine receptors, causing the voltage-gated Na^+^ channels to close. While the resting potential is quickly restored by the operation of the Cl^-^ channels and leak K^+^ channels [19], Nka is crucial to the homeostatic restoration of the Na^+^ and K^+^ concentration gradients [20], providing the chemical potential energy for the generation of the action potential which is the basis of electrogenesis.

### Molecular characterization of the deduced Nkaα sequences from *E*. *electricus*

We report for the first time the expression of four *nkaα* isoforms (*nkaα1c1*, *nkaα1c2*, *nkaα2* and *nkaα3*) in the SM and EOs of *E*. *electricus*. Based on homology modelling of human NKAα [38], the amino acid residues involved in the binding of 3 Na^+^ and 2 K^+^ were deduced from the four translated Nkaα of *E*. *electricus*. For Na^+^ binding, site I was formed entirely by side chain oxygen atoms of residues in three helices (TM5, TM6 and TM8). Site II was formed approximately on the TM4 helix with three main chain carbonyls plus four side chain oxygen atoms (Asp 831 and Asp 835 on TM6 and Glu 353 on TM4). Site III was formed by the carbonyls of Gly 833 and Thr 834 in TM6, hydroxyl of Tyr 798 in TM5, and the carboxyl of Glu 981 in TM9. For K^+^ binding, site I was formed at a position very similar to the binding site I for Na^+^ but with the involvement of Ser 802, while site II was also formed on the TM4 helix with the K^+^ site shifted toward the extracellular side by a turn of the α-helix. Morth and co-workers [39] studied the functional importance of the C-terminus of Nka by deleting the five residues at the C-terminus. The truncated enzyme (delKETYY) exhibited an extraordinary 26-fold reduction in Na^+^ affinity. Nkaα1c2, Nkaα2 and Nkaα3 of *E*. *electricus* possessed the KETYY motif while the lysine residue for Nkaα1c1 was replaced by an arginine residue (i.e. RETYY). This might affect the Na^+^ binding affinity of Nkaα1c1 in *E*. *electricus*.

During the catalytic cycle of P-type ATPases, the P domain interacts with the N domain and the A domain, leading to auto-phosphorylation and dephosphorylation, respectively. The phosphorylation site is located in the aspartate residue of a conserved Asp-Lys-Thr-Gly-Thr (DKTGT) motif that is found in all P-type ATPases. This conserved motif was also found in all the four Nkaα-subunits of *E*. *electricus*, and thus confirming the importance of this phosphorylation site. Furthermore, two other sequence motifs, Thr-Gly-Asp (TGD) and Thr-Gly-Asp-Gly-X-Asn-Asp (TGDGXND), which are involved in Mg^2+^ coordination associated with ATP binding at the phosphorylation site [40], were also present in all the four Nkaα-subunits of *E*. *electricus*. Xu [41] showed that the native activity of NKA was markedly elevated when protein-protein interaction occurred at the extracellular DVEDSYGQQWTYEQR (D-R) region in the α-subunit of the enzyme. Furthermore, Xu [41] demonstrated that the activation of NKA did not only protect enzyme function against denaturation, but also directly affected cellular activities by regulating intracellular Ca^2+^ transients and inducing a positive inotropic effect in isolated rat cardiac myocytes. A comparison between the D-R regions of various NKAα-subunits from *E*. *electricus* and those from rat revealed that they were highly conserved, suggesting that the D-R region might be a universal activation site for all the NKAα/Nkaα subunits.

Both cAMP-dependent PKA and PKC can be involved in the phosphorylation of NKAα [42,43]. The target site of PKA phosphorylation is the serine residue at position 963 (according to the alignment in Fig. 1) and it is apparently conserved in all the four Nkaα subunits of *E*. *electricus*. Beguin et al. [44] identified Thr10 and Ser11 as PKC phosphorylation sites in the Nka of *Bufo marinus* by site-directed mutagenesis. For *E*. *electricus*, Thr10 (corresponding to Thr31 in the alignment in Fig. 1) and Ser11 (corresponding to Ser32) were identified in Nkaα1c1 and Nkaα1c2. However, in Nkaα2 of *E*. *electricus*, Thr31 was substituted by a serine residue, and in Nkaα3, Ser32 was substituted by a threonine residue.

Jørgensen and Pedersen [45] demonstrated that the substitution of Thr781 in TM5 of NKAα1 with a serine residue increased the Gibbs free energy for Na^+^ and K^+^ binding. In the case of *E*. *electricus*, this substitution was observed in Nkaα1c2 and Nkaα2, indicating that their affinities to Na^+^ and K^+^ were probably different from those of Nkaα1c1 and Nkaα3.

### Differential expression of *nkaα1c1* and *nkaα2* between the SM and the three EOs

Based on mRNA expression levels, the major *nkaα* isoforms expressed in the SM and the three EOs of juvenile and adult *E*. *electricus* were *nkaα1c1* and *nkaα2*, and they were differentially expressed among the SM and the EOs. In mammals, NKAα isoforms have different tissue distributions [22,46], different affinities for ouabain and substrate ions [22,47], and differential regulation via hormones and protein kinases [48]. NKAα1 is found in nearly every tissue, but the other isoforms are more limited in expression [49]. Adult mammalian SMs express NKAα2 as the major isoform, at up to 87% of the total NKAα subunit content, with NKAα1 being the minor isoform [44,50]. In zebrafish (*Danio rerio*), Nkaα2 is expressed in every tissue except the testis [51]. In other teleost fishes such as *Fundulus heteroclitus* [52], *O*. *mykiss* [53] and *Trematomus bernacchii* [54], Nkaα2 is found in the brain, muscle, and in the case of *O*. *mykiss*, in the eye as well. By contrast, *nkaα1c1* appeared to be SM-predominant in *E*. *electricus*, as its mRNA expression level was higher in the muscle than in the three EOs, while *nkaα2* appeared to be EO-predominant as it was mainly expressed in the EOs. The development of EOs in *E*. *electricus* happens very soon after birth, with SM and EOs having the same embryonic origin. It has been reported that fish as small as 15 mm have begun EO development [55,56]. The initial growth of a weak EO allows for orientation and strong EOs start to develop when *E*. *electricus* is approximately 40 mm. Therefore, our results indicate for the first time a foundational change in the expression of *nkaα* isoforms during embryonic development of SM and EOs in *E*. *electricus*.

Also our results indicate for the first time that the mRNA expression levels of *nkaα2* in the EOs of adult fish were substantially higher than those in the EOs of juvenile fish. While the expression level of *nkaα2* was the highest in the Hunter’s EO of juvenile fish, it was the highest in the main EO of adult fish. Furthermore, the differences in expression levels of *nkaα2* between the EOs of adult fish and the corresponding EOs of juvenile fish were two orders of magnitude greater than the difference in expression level of *nkaα1c1* between the SM of adult fish and the SM of juvenile fish. These results indicate that, besides increasing in the number of electrocytes, there are changes at the molecular level during the growth and development of the three EOs, especially for the main EO as it becomes more powerful in adult fish. Based on the mRNA expression levels of *nkaα2* in adult fish, it is apparent that there are molecular differences among the three EOs besides differences in the density of packing of electrocytes as reported previously [19]. However, it must be emphasized that the observed differences in mRNA expression levels of *nkaα* may or may not match the differences in protein levels of Nkaα or enzyme activities of Nka among the EOs as there could be additional post-transcriptional and/or post-translational processes regulating protein abundance and activity.

In mammalian brains, three isoforms, *NKAα1*, *NKAα2* and *NKAα3*, have been identified [22,57]. These three NKAα-subunits differ with regard to their Na^+^, K^+^ and ATP sensitivity [58,59]. NKAα2 is an astrocyte-specific isoform, and astrocytes are responsible for the clearance of K^+^ from the extracellular fluid after neuronal activity [60]. The expression of various rat NKAα-subunits in *Xenopus* oocytes reveals that NKAα2 has a three-fold higher sensitivity to extracellular Na^+^ when compared with NKAα1 and NKAα3, causing NKAα2 to be more sensitive to the membrane potential [61]. These could be the reasons why *nkaα2* was the predominant *nkaα* isoform expressed in the EOs of *E*. *electricus*.

Besides *nkaα2*, the EOs of juvenile and adult *E*. *electricus* had relatively low levels of *nkaα1c1* and *nkaα1c2* expression. Of note, Lowe et al. [25] found that the sensitivity of Nka ouabain site differed by a 1.6-fold affinity from the innervated and non-innervated electrocyte membrane in the main EO of *E*. *electricus*. Using immunofluorescence microscopy and commercially available antibodies raised against mammalian NKA, they found that this difference in sensitivity was due to the polarization of two different forms of Nkaα, with Nkaα1 localized to the innervated membrane and Nkaα2 localized to the non-innervated membrane [25]. As the innervated and non-innervated membranes are known to differ in terms of their specific resistance [2], the difference in Nkaα distribution may be related to differences in Na^+^ or K^+^ permeability between these two membranes. As our result indicate the presence of two *nkaα1* isoforms, it would be essential to examine the protein expression and localization of Nkaα1c1 and Nkaα1c2 in the innervated membrane of the three EOs of *E*. *electricus* in the future.

### Variation in protein abundance of Nkaα among the SM and the three EOs

EOs comprise electrocytes containing relatively massive quantities of Na^+^ channels for their specialized function, and therefore require proportionally large amounts of Nka to maintain ionic gradients. Indeed, Nkaα protein was barely detectable in the SM, but was strongly detected in the Hunter’s EO and the main EO, with the protein abundance of Nkaα in the former significantly greater than that of the latter, and weakly expressed in the Sach’s EO, of juvenile *E*. *electricus*. In comparison, there was an apparent change in the Nkaα expression pattern among the three EOs of adult *E*. *electricus*; the protein abundance of Nkaα in the main EO was significantly higher than that in the Hunter’s EO, corroborating the results on *nkaα2* mRNA expression. These results confirmed that the expression of *nkaα*/Nkaα in the main EO and the Hunter’s EO were regulated both transcriptionally and translationally during the growth of *E*. *electricus*. As in juvenile fish, the Sach’s EO of adult fish had very low Nkaα expression. The Sach’s EO produces low voltage EODs (~10 V) at a frequency of up to 25 Hz, while the main EO and the Hunter’s EO produces high voltages (~500 V) at a rate of up to several hundred Hz [10], and there is a higher density of transmembrane Na^+^ channel in electrocytes of the main EO than in those of the Sach’s EO [11]. This could explain why the Sach’s EO had a relative low protein abundance of Nkaα. Incidentally, through western blotting using the anti-NKA α5 antibody, Gallant et al. [62] demonstrated that the protein abundance of Nkaα in the SM and the EO of the weakly electric mormyrid, *Brienomyrus brachyistius*, were approximately equal. Furthermore, they reported that two forms of Nkaα were detected in the SM (99 and 90 kDa) whereas only one form (90 kDa) was expressed in the EO of *B*. *brachyistius*. When taken together with results obtained from the Sach’s EO of juvenile and adult *E*. *electricus*, it would appear that an EO generating weak EODs can maintain the Na^+^ and K^+^ gradients upon discharge with an Nkaα protein expression level comparable to that of SM, albeit involving different isoforms of Nkaα.

### Differences in kinetic properties of Nka among the three EOs

Due to the similar protein abundance of Nkaα, Gallant et al. [62] proposed, but without evidence, that there could be differences in the efficiency/kinetic properties of Nka between the SM and the EO of *B*. *brachyistius*. Here, we report for the first time that for juvenile *E*. *electricus*, the *V*_sat_ of Nka from the SM was significantly lower than those of Nka from the main EO, the Hunter’s EO and the Sach’s EO. More importantly, our results indicated that there were significant differences in kinetic properties of Nka among the three EOs of juvenile *E*. *electricus*. Firstly, in agreement with *V*_sat_ results, the highest and lowest *V*_max_ of Nka were detected in the main EO and the Sach’s EO, respectively, with the Hunter’s EO having a *V*_max_ value intermediate between the two. This is in agreement with the fact that the main EO produces high voltage EODs, the Sach’s EO produces low voltage EODs, and the Hunter’s EO functions intermediately between the main EO and the Sach’s EO [2,10]. As ion pumps impose significant demands on the metabolic resources of excitable tissues [63], our results indicate that the metabolic costs of EOD is the highest in the main EO, followed by the Hunter’s EO and the Sach’s EO, in *E*. *electricus*. Secondly, the Nka from the main EO had the lowest *K*_*m*_ (or highest affinity) for Na^+^ and K^+^ among the three EOs. As it has been reported that the main EO of *E*. *electricus* exhibits an outwardly directed voltage-gated K^+^ current in a small fraction of patches on the innervated membrane of its electrocytes [11], our results indicate that the Nka of the main EO might be more effective in clearing K^+^ from the extracellular fluid after EOD than the Nka of the Hunter’s EO and the Sach’s EO.

For juvenile *E*. *electricus*, in spite of the Nkaα protein abundance in the Hunter’s EO being significantly higher than that in the main EO, the *V*_Sat_ and *V*_max_ of Nka from these two EOs were comparable to each other. Of note, NKA/Nka is a dimer of α- and β-subunits, whereby the β-subunit is essential to the stability of the α-subunit and its delivery and insertion into the plasma membrane [64]. In addition, NKAβ/Nkaβ can modulate the affinity of NKAα/Nkaα to Na^+^ and K^+^, and alter the NKA/Nka activity. As there are three isoforms of the β-subunit [65], our results suggest that various isoforms of *nkaβ*/Nkaβ might be differentially expressed in the main EO and the Hunter’s EO of *E*. *electricus*.

## Conclusion

To date, only one *nkaα* with an unknown isoform identity has been sequenced from the electric organ of *E*. *electricus* (AF356351) [26], and we had verified it to be Nkaα2. In addition, we report the expression of *nkaα1c1*, *nkaα1c2* and *nkaα3* in the SM and the EOs of *E*. *electricus*. The major *nkaα* isoforms expressed in the SM and the three EOs were *nkaα1c1* and nkaα2, respectively. Molecular characterization of the deduced Nkaα1c1 and Nkaα2 sequences indicated that they probably had different affinities to Na^+^ and K^+^. Western blotting results showed that Nkaα was barely detectable in the SM. By contrast, in line with the electric properties of the three EOs, Nkaα was strongly detected in the main EO and the Hunter’s EO, and weakly expressed in the Sach’s EO of *E*. *electricus*. More importantly, the highest and lowest *V*_max_ of Nka were detected in the main EO and the Sach’s EO, respectively, with the Hunter’s EO having a *V*_max_ value intermediate between the two. Furthermore, the Nka from the main EO had the lowest *K*_*m*_ (or highest affinity) for Na^+^ and K^+^ among the three EOs, suggesting that the Nka of the main EO was more effective than those of the Hunter’s EO and the Sach’s EO in clearing K^+^ from the extracellular fluid and maintaining intracellular Na^+^ and K^+^ homeostasis after EO discharge. As the Nkaβ subunit inserts and anchors the Nkaαβ complex in the plasma membrane and regulates the Na^+^ and K^+^ affinities [23], it would be essential to examine the possible differences in expression of *nkaβ*/Nkaβ isoforms among the three EOs of *E*. *electricus* in future studies.

## References

[1] Nelson ME. Electric fish. Curr Biol. 2011; 21: R528–R529. 10.1016/j.cub.2011.03.045 21783026

[2] Bennett MVL. Electric organs. In: Hoar WS, Randal DJ, editors. Fish Physiology. New York: Academic Press; 1971 pp. 346–491.

[3] Valasco T. *Electrophorus electricus* Animal Diversity Web: The Regents of the University of Michigan. 2003. Available: http://animaldiversity.ummz.umich.edu/accounts/Electrophorus_electricus/. Accessed 7 April 2014.

[4] Berra TM. Freshwater Fish Distribution. San Diego: Academic Press; 2001.

[5] Grundfest H. A four-factor ionic hypothesis of spike electrogenesis. Biol Bull. 1960; 119: 284.

[6] Albert JS, Crampton WGR. Electroreception and Electrogenesis In: Evans DH, Claiborne JB, editors. The Physiology of Fishes. Boca Raton: CRC Press; 2005 pp. 431–472.

[7] Szabo T. The origin of electric organs of *Electrophorus electricus* . Anat Rec. 1966;155: 103–110. 6006796 10.1002/ar.1091550112

[8] Couceiro A, Fessard A. Quelques données histologiques sur le noyau de commande centrale de la décharge électrique chez l'*Electrophorus electricus* . Neurobiologia. 1953;16: 299–305.

[9] Keynes RD, Martins-Ferreira H. Membrane potentials in the electroplates of the electric eel. J Physiol. 1953;119: 315–351. 13035755 10.1113/jphysiol.1953.sp004849 PMC1392804

[10] Ortega H, Vari RP. Annotated checklist of the freshwater fishes of Peru Smithsonian Contrib Zool. 1986;437: 1–25.

[11] Shenkel S, Sigworth F. Patch recordings from the electrocytes of *Electrophorus electricus*. Na currents and P_Na_/P_K_ variability. J Gen Physiol 1991;97: 1013–1041. 1650809 10.1085/jgp.97.5.1013 PMC2216506

[12] Keynes KD. The development of the electric organ in *Electrophorus electricus* In: Chagas C, Paes de Carvalho A, editors. Bioelectrogenesis. New York: Elsevier; 1961 pp. 14–18.

[13] Machado RD, De Souza W, Benchimol M, Attias M, Porter K. Observations on the innervated face of the electrocyte of the main organ of the electric eel (*Electrophorus electricus* L.). Cell Tissue Res. 1980;213: 69–80. 7459996 10.1007/BF00236921

[14] Machado RD, de Souza W, Cotta-Pereira G, de Oliveira Castro G. On the fine structure of the electrocyte of *Electrophorus electricus* L. Cell Tissue Res. 1976;174: 355–366. 1000580 10.1007/BF00220681

[15] Nakamura Y, Nakajima S, Grundfest H. Analysis of spike electrogenesis and depolarizing K inactivation in electroplaques of *Electrophorus electricus* L. J Gen Physiol. 1965;49: 321–349. 19873566 10.1085/jgp.49.2.321 PMC2195482

[16] Nachmansohn D. Proteins in excitable membranes. Science. 1970;168: 1059–1066. 4392552 10.1126/science.168.3935.1059

[17] Somló C., De Souza W., Machado R, Hassón-Voloch DA. Biochemical and cytochemical localization of ATPases on the membranes of the electrocyte of *Electrophorus electricus* . Cell Tissue Res. 1977;185: 115–128. 145320 10.1007/BF00226673

[18] Ellisman MH, Levinson SR. Immunocytochemical localization of sodium channel distributions in the excitable membranes of *Electrophorus electricus* . PNAS. 1982;79: 6707–6711. 6292913 10.1073/pnas.79.21.6707 PMC347198

[19] Gotter AL, Kaetzel MA, Dedman JR. *Electrophorus electricus* as a Model System for the Study of Membrane Excitability. Complementary Biochem Physiol. 1998;119: 225–241. 11253789 10.1016/s1095-6433(97)00414-5

[20] Horisberger J, Lemas V, Kraehenbuhl J, Rossier BC. Structure-Function Relationship of Na, K-ATPase. Annu Rev Physiol. 1991;53: 565–584. 1645948 10.1146/annurev.ph.53.030191.003025

[21] Jørgensen PL, Håkansson KO, Karlish SJ. Structure and mechanism of Na, K-ATPase: functional sites and their interactions. Annu Rev Physiol. 2003;65: 817–849. 12524462 10.1146/annurev.physiol.65.092101.142558

[22] Blanco G, Mercer RW. Isozymes of the Na-K-ATPase: heterogeneity in structure, diversity in function. Am J Physiol Renal Physiol. 1998;275: F633–F650. 9815123 10.1152/ajprenal.1998.275.5.F633

[23] Skou J, Esmann M. The Na, K-ATPase. J Bioenerg Biomembr. 1992;24: 249–261. 1328174 10.1007/BF00768846

[24] Lingrel JB, Williams MT, Vorhees CV, Moseley AE. Na, K-ATPase and the role of α isoforms in behavior. J Bioenerg Biomembr. 2007;39: 385–389. 18044013 10.1007/s10863-007-9107-9

[25] Lowe J, Araujo GMN, Pedrenho AR, Nunes-Tavares NL, Ribeiro MG, Hassón-Voloch A. Polarized distribution of Na^+^, K^+^-ATPase a-subunit isoforms in electrocyte membranes. Biochim Biophys Acta. 2004;1661: 40–46. 14967473 10.1016/j.bbamem.2003.11.020

[26] Kaya S, Yokoyama A, Imagawa T, Taniguchi K, Froehlich JP, Albers RW. Cloning of the eel electroplax Na^+^, K^+^-ATPase alpha subunit. Ann N Y Acad Sci. 1997;834: 129–131. 9405797 10.1111/j.1749-6632.1997.tb52238.x

[27] Hall TA. BioEdit: a user-friendly biological sequence editor and analysis program for Windows 95/98/NT. Nucl Acids Symp Ser. 1999;41: 95–98.

[28] McGuffin LJ, Bryson K, Jones DT. The PSIPRED protein structure prediction server. Bioinformatics. 2000;16: 404–405. 10869041 10.1093/bioinformatics/16.4.404

[29] Felsenstein J. PHYLIP—Phylogeny Inference Package (Version 3.2). Cladistics. 1989;5: 164–166.

[30] Wong ML, Medrano JF. Real-time PCR for mRNA quantitation. Biotechniques. 2005;39: 75–85. 16060372 10.2144/05391RV01

[31] Gerwick L, Corley-Smith G, Bayne CJ. Gene transcript changes in individual rainbow trout livers following an inflammatory stimulus. Fish Shellfish Immunol. 2007;22: 157–171. 16762566 10.1016/j.fsi.2006.04.003

[32] Laemmli UK. Cleavage of structural proteins during the assembly of the head bacteriophage T4. Nature. 1970;227: 680–685. 5432063 10.1038/227680a0

[33] Wilson JM. The use of immunochemistry in the study of branchial ion transport mechanisms In: Baldisserotto B, Romero JMM, Kapoor BG, editors. Fish Osmoregulation. Enfield, NH: Science Publishers; 2007 pp. 359–394.

[34] Ip YK, Loong AM, Kuah JS, Sim EW, Chen XL, Wong WP, et al Roles of three branchial Na^+^-K^+^-ATPase α-subunit isoforms in freshwater adaptation, seawater acclimation, and active ammonia excretion in *Anabas testudineus* . Am J Physiol Regul Integr Comp Physiol. 2012;303: R112–R125. 10.1152/ajpregu.00618.2011 22621969

[35] Bradford MM. A rapid and sensitive method for quantitation of microgram quantities of protein utilising the principle of protein-dye binding. Anal Biochem. 1976;72: 248–254. 942051 10.1016/0003-2697(76)90527-3

[36] Bennett MV, Sandri C. The electromotor system of the electric eel investigated with horseradish peroxidase as a retrograde tracer. Brain Res. 1989;488: 22–30. 2743117 10.1016/0006-8993(89)90689-6

[37] Fritz LC, Brockes JP. Immunochemical properties and cytochemical localization of the voltage-sensitive sodium channel from the electroplax of the eel (*Electrophorus electricus*). J Neurosci. 1983;3: 2300–2309. 6313876 10.1523/JNEUROSCI.03-11-02300.1983 PMC6564629

[38] Ogawa H, Toyoshima C. Homology modeling of the cation binding sites of Na^+^ K^+^-ATPase. PNAS. 2002;99: 15977–15982. 12461183 10.1073/pnas.202622299 PMC138550

[39] Morth JP, Pedersen BP, Toustrup-Jensen MS, Sørensen TL-M, Petersen J, Andersen JP, et al Crystal structure of the sodium—potassium pump. Nature. 2007;450: 1043–1049. 18075585 10.1038/nature06419

[40] Palmgren MG, Nissen P. P-type ATPases. Annu Rev Biophys. 2011;40: 243–266. 10.1146/annurev.biophys.093008.131331 21351879

[41] Xu KY. Activation of (Na^+^, K^+^)-ATPase. Biochem Biophys Res Commun. 2005;338: 1669–1677. 16263081 10.1016/j.bbrc.2005.10.067

[42] Middleton JP, Khan W, Collinsworth G, Hannun Y, Medford R. Heterogeneity of protein kinase C-mediated rapid regulation of Na/K-ATPase in kidney epithelial cells. J Biol Chem. 1993;268: 15958–15964. 8393456

[43] Aperia A, Holtback U, Syren ML, Svensson LB, Fryckstedt J, Greengard P. Activation/deactivation of renal Na^+^, K^+^-ATPase: a final common pathway for regulation of natriuresis. FASEB J. 1994;8: 436–439. 8168694 10.1096/fasebj.8.6.8168694

[44] Beguin P, Beggah AT, Chibalin AV, Burgener-Kairuz P, Jaisser F, Mathews PM, et al Phosphorylation of the Na, K-ATPase alpha-subunit by protein kinase A and C in vitro and in intact cells. Identification of a novel motif for PKC-mediated phosphorylation. J Biol Chem. 1994;269(39): 24437–24445. 7929106

[45] Jørgensen PL, Pedersen PA. Structure—function relationships of Na^+^, K^+^, ATP, or Mg^2+^ binding and energy transduction in Na, K-ATPase. Biochim Biophys Acta. 2001;1505: 57–74. 11248189 10.1016/s0005-2728(00)00277-2

[46] Orlowski J, Lingrel JB. Tissue-specific and developmental regulation of rat Na, K-ATPase catalytic α isoform and β subunit mRNAs. J Biol Chem. 1988;263: 10436–10442. 2839491

[47] Lücking K, Nielsen JM, Pedersen PA, Jørgensen PL. Na-K-ATPase isoform (alpha 3, alpha 2, alpha 1) abundance in rat kidney estimated by competitive RT-PCR and ouabain binding. Am J Physiol. 1996;271(2): F253–F260. 8770155 10.1152/ajprenal.1996.271.2.F253

[48] Therien AG, Blostein R. Mechanisms of sodium pump regulation. Am J Physiol Cell Physiol. 2000;279(3): C541–C566. 10942705 10.1152/ajpcell.2000.279.3.C541

[49] Lingrel J. Na,K-ATPase: Isoform structure, function, and expression. J Bioenerg Biomembr. 1992;24: 263–270. 1328175 10.1007/BF00768847

[50] He S, Shelly DA, Moseley AE, James PF, James JH, Paul RJ, et al The α1 and α2 isoforms of Na-K-ATPase play different roles in skeletal muscle contractility. Am J Physiol Regul Integr Comp Physiol. 2001;281: R917–R925. 11507009 10.1152/ajpregu.2001.281.3.R917

[51] Rajarao SJR, Canfield VA, Mohideen M-AP, Yan Y-L, Postlethwait JH, Cheng KC, et al The repertoire of Na, K-ATPase α and β subunit genes expressed in the zebrafish, *Danio rerio* . Genome Res. 2001;11: 1211–1220. 11435403 10.1101/gr.186001 PMC311090

[52] Semple JW, Green HJ, Schulte PM. Molecular cloning and characterization of two Na/K-ATPase isoforms in *Fundulus heteroclitus* . Mar Biotechnol (NY). 2002;4:512–519. 14961245 10.1007/s10126-002-0031-z

[53] Richards JG, Semple JW, Bystriansky JS, Schulte PM. Na^+^/K^+^-ATPase α-isoform switching in gills of rainbow trout (*Oncorhynchus mykiss*) during salinity transfer. J Exp Biol. 2003;206: 4475–4486. 14610032 10.1242/jeb.00701

[54] Guynn S, Scofield M, Petzel D. Identification of mRNA and protein expression of the Na/K-ATPase α1-, α2-and α3-subunit isoforms in Antarctic and New Zealand nototheniid fishes. J Exp Mar Bio Ecol. 2002;273: 15–32.

[55] Keynes R. Electric Organs In: Brown M Brown, editor. The Physiology of Fishes, Volume II New York: Academic Press, Inc.; 1957 pp. 323–343.

[56] Moller P. Electric Fishes: History and Behavior. New York: Chapman & Hall; 1995.

[57] Taguchi K, Kumanogoh H, Nakamura S, Maekawa S. Ouabain-induced isoform-specific localization change of the Na^+^, K^+^-ATPase α subunit in the synaptic plasma membrane of rat brain. Neurosci Lett. 2007;413(1): 42–45. 17207930 10.1016/j.neulet.2006.11.061

[58] Jewell EA, Lingrel JB. Comparison of the substrate dependence properties of the rat Na, K-ATPase alpha 1, alpha 2, and alpha 3 isoforms expressed in HeLa cells. J Biol Chem. 1991;266: 16925–16930. 1653250

[59] Zahler R, Zhang ZT, Manor M, Boron WF. Sodium kinetics of Na, K-ATPase alpha isoforms in intact transfected HeLa cells. J Gen Physiol. 1997;110: 201–213. 9236212 10.1085/jgp.110.2.201 PMC2233788

[60] Peng L, Martin-Vasallo P, Sweadner KJ. Isoforms of Na, K-ATPase alpha and beta subunits in the rat cerebellum and in granule cell cultures. J Neurosci. 1997;17: 3488–502. 9133374 10.1523/JNEUROSCI.17-10-03488.1997 PMC6573685

[61] Horisberger J, Kharoubi-Hess S. Functional differences between α subunit isoforms of the rat Na, K-ATPase expressed in *Xenopus* oocytes. J Physiol. 2002;539: 669–680. 11897839 10.1113/jphysiol.2001.013201 PMC2290179

[62] Gallant JR, Hopkins CD, Deitcher DL. Differential expression of genes and proteins between electric organ and skeletal muscle in the mormyrid electric fish *Brienomyrus brachyistius* . J Exp Biol. 2012;215(14): 2479–2494. 10.1242/jeb.063222 22723488 PMC3379852

[63] Clausen T, Van Hardeveld C, Everts ME. Significance of cation transport in control of energy metabolism and thermogenesis. Physiol Rev. 1991;71: 733–773. 2057526 10.1152/physrev.1991.71.3.733

[64] Sweadner KJ. Overlapping and diverse distribution of Na-K ATPase isozymes in neruons and glia. Can J. Physiol PHarmacol. 1992;70: S255–S259. 1338295 10.1139/y92-269

[65] Sweadner KJ. Isozymes of the Na^+^/K^+^-ATPase. Biochim Biophys Acta. 1989;988: 185–220. 2541792 10.1016/0304-4157(89)90019-1

